# Validity, reliability, and acceptability of the Evidence-Informed Decision-Making (EIDM) competence measure

**DOI:** 10.1371/journal.pone.0272699

**Published:** 2022-08-05

**Authors:** Emily Belita, Kathryn Fisher, Jennifer Yost, Janet E. Squires, Rebecca Ganann, Maureen Dobbins

**Affiliations:** 1 School of Nursing, McMaster University, Hamilton, Ontario, Canada; 2 M. Louise Fitzpatrick College of Nursing, Villanova University, Villanova, Pennsylvania, United States of America; 3 School of Nursing/École des Sciences Infirmières, University of Ottawa/Université d’Ottawa, Ottawa, Ontario, Canada; 4 National Collaborating Centre for Methods and Tools, McMaster University, Hamilton, Ontario, Canada; Georgia Southern University, UNITED STATES

## Abstract

Valid, reliable, and acceptable tools for assessing self-reported competence in evidence-informed decision-making (EIDM) are required to provide insight into the current status of EIDM knowledge, skills, attitudes/beliefs, and behaviours for registered nurses working in public health. The purpose of this study was to assess the validity, reliability, and acceptability of the EIDM Competence Measure. A psychometric study design was employed guided by the Standards for Educational and Psychological Testing and general measurement development principles. All registered nurses working across 16 public health units in Ontario, Canada were invited to complete the newly developed EIDM Competence Measure via an online survey. The EIDM Competence Measure is a self-reported tool consisting of four EIDM subscales: 1) knowledge; 2) skills; 3) attitudes/beliefs; and 4) behaviours. Acceptability was measured by completion time and percentage of missing data of the original 40-item tool. The internal structure of the tool was first assessed through item-subscale total and item-item correlations within subscales for potential item reduction of the original 40-item tool. Following item reduction which resulted in a revised 27-item EIDM Competence Measure, a principal component analysis using an oblique rotation was performed to confirm the four subscale structure. Validity based on relationships to other variables was assessed by exploring associations between EIDM competence attributes and individual factors (e.g., years of nursing experience, education) and organizational factors (e.g., resource allocation). Internal reliability within each subscale was analyzed using Cronbach’s alphas. Across 16 participating public health units, 201 nurses (mean years as a registered nurse = 18.1, predominantly female n = 197; 98%) completed the EIDM Competence Measure. Overall missing data were minimal as 93% of participants completed the entire original 40-item tool (i.e., no missing data), with 7% of participants having one or more items with missing data. Only one participant (0.5%) had >10% of missing data (i.e., more than 4 out of 40 items with data missing). Mean completion time was 7 minutes and 20 seconds for the 40-item tool. Extraction of a four-factor model based on the 27-item version of the scale showed substantial factor loadings (>0.4) that aligned with the four EIDM subscales of knowledge, skills, attitudes/beliefs, and behaviours. Significant relationships between EIDM competence subscale scores and education, EIDM training, EIDM project involvement, and supportive organizational culture were observed. Cronbach’s alphas exceeded minimum standards for all subscales: knowledge (α = 0.96); skills (α = 0.93); attitudes/beliefs (α = 0.80); and behaviours (α = 0.94).

## Introduction

The crucial need for implementing evidence-informed public health interventions that are effective and cost-efficient is increasingly demonstrated amid emerging communicable diseases, sustained rates of chronic conditions, climate change, and other public health threats to populations, communities, and individuals [1]. The concept of basing public health decision making on diverse forms of high quality, available evidence is recognized as evidence-informed decision-making (EIDM) [2]. EIDM in public health practice involves the identification, appraisal, and application of evidence related to research, along with professional expertise, local context, and client and community characteristics, needs, and preferences [2–4].

Across the functions of public health preparedness, prevention, protection, and promotion [5], there is considerable evidence of high-quality, effective, and cost-efficient interventions [6, 7]. Despite this evidence base, a persistent gap exists where research evidence is not consistently used by public health professionals to inform decision-making in practice [8, 9]. Findings across multiple studies highlight this research-to-practice gap among the public health workforce, of which nurses are the largest professional discipline [10]. In two studies exploring EIDM capacity building among public health professionals, participants reported that only 58–72% of public health programs offered by their state and local organizations were informed by research evidence [11, 12]. Furthermore, in a review of 33 studies exploring public health professionals’ information needs for evidence-informed decision-making, Barr-Walker [13] reported that colleagues served as a primary information source more often than online databases. In another study investigating public health decision-making among local governments in Australia, [14] published literature such as journal articles or reports by academic institutions were reported to be the least influential and least useful resources in decision-making. Findings from international studies centred specifically on nurses in public health further validate this research-to-practice gap. Authors of studies in Canada [15] and Norway [16] have reported that nurses frequently used knowledge from clients, other clinical experts, and professional development trainings to inform their professional practice, and in fewer instances relied on evidence from published research.

Deficits in EIDM public health practice relate largely to low levels of confidence, knowledge, and skills in performing EIDM related tasks [17, 18], along with organizational barriers, such as work cultures resistant to change, insufficient resource allocation, or lack of protected time for EIDM work [17, 19]. Strategies to encourage sustained organization-wide EIDM uptake include supportive nursing leadership and mentorship, a focus on competence development at the individual practitioner level through professional development opportunities [17, 20], integration of EIDM as a strategic priority in organizational missions or visions, and explicit indicators of EIDM responsibilities and expectations for practitioners and health care leaders [20–22]. Regarding the latter, the articulation of EIDM expectations provides organizations and health care professionals with shared and consistent language around standards that can be integrated into competence assessment measures for use in real-world practice.

While many tools exist to measure EIDM, there are only a few that specifically seek to assess EIDM competence among health care professionals. The Evidence Based Practice Evaluation Competence Questionnaire (EBP-COQ) is a 25-item tool with demonstrated internal reliability, construct and discriminant validity which assesses self-reported competence in EIDM among undergraduate nursing students [23]. Developers of this instrument defined competence as the ability to choose and use knowledge, skills and attitudes with the intention of performing a task in a specific context [23]. The Fresno test has also been labelled by its developers as a competence assessment measure for EIDM knowledge and skills [24]. This objective measure has been tested among family practice residents, EIDM educators [24], and an adapted version used among pediatric nurses [25]. The Fresno test requires responses to short answer questions based on hypothetical clinical scenarios. Inter-rater reliability, internal consistency, and content, and construct validity have all been established through its psychometric testing [24]. While the original developers do not explicitly discuss their established conceptual definition of competence [24], the author of the adapted Fresno test discusses the conceptual meaning of competence as related to knowledge and skill acquisition [25].

Missing from the conceptual definitions of competence used in these existing measures is the inclusion of all attributes that comprise competence, which includes knowledge, skills, attitudes/values, and behaviours [26–28]. Competence has been defined as the integration of these four attributes with a focus on the quality of task performance related to a specific standard [26, 28, 29]. Specifically, in relation to competence in EIDM, these four attributes are well described across the literature (see Table 1).

**Table 1:** EIDM competence attribute definitions.

Knowledge	Skills	Attitudes/values	Behavioural
Understanding the principles, steps, and practical aspects of EIDM [30–33]	Applying knowledge of EIDM steps, principles in a practical setting (e.g., clinical case scenario) [30, 31, 33, 34]	Views, perceptions, beliefs, thoughts, intentions about agreement, acceptance related to EIDM overall or its aspects [30, 32, 33]	Performance of EIDM tasks in a real-world setting (e.g., searching databases for evidence based real life clinical problem) [30, 33, 34]

This conceptual underrepresentation of EIDM competence attributes has also been confirmed in a recent systematic review [35] of 35 measures assessing EIDM knowledge, skills, attitudes, and/or behaviours in which the authors reported that only three measures assessed all four competence attributes: a) the Evidence-Based Practice Questionnaire [36], b) the School Nursing Evidence-Based Practice Questionnaire [37], and c) a self-developed measure by Chiu et al. [38]. In addition, there were limitations among these three measures, including a lack of assessment of the quality of the competence attributes, a critical component of competence, particularly with respect to EIDM behaviour items [35].

To address these conceptual gaps among EIDM measures, this study aimed to develop and psychometrically test a comprehensive self-report EIDM competence measure assessing the quality of knowledge, skills, attitudes/beliefs, and behaviours among nurses in public health. The first phase of this study, described elsewhere [39], focused on the process of item development, content validation (analyzing the relationship between test content and the construct under measure) [40], and response process assessment (exploring how thought processes used in responding to a scale align with a construct under measure) [40] of the new EIDM Competence Measure. The purpose of this paper is to describe the second phase of the study of the EIDM Competence Measure; to assess acceptability, internal reliability, and validity evidence, based on the internal structure and relationship of items in the measure to other variables.

## Methods

### Design

A psychometric study design was used for this study. It was guided by general measure development principles [41] and the Standards for Educational and Psychological Testing which provide established criteria for the development and psychometric evaluation of tests, with a focus on assessment of validity evidence [40]. Full ethical approval was granted for the study by the Hamilton Integrated Research Ethics Board.

### Sample and recruitment

Recruitment occurred from November 2019 to March 2020. A multi-stage sampling design was used; health units were recruited at the first stage and individual nurses were recruited at the second stage. A convenience sample of public health units in Ontario was recruited. Existing relationships with Medical Officers of Health were used to identify organizations for this convenience sample. Thirty-two Ontario health units were invited to participate through an email sent to each Medical Officer of Health informing them about and requesting support for the study. Champions (e.g., Chief Nursing Officers, nursing managers) were identified by Medical Officers of Health and responsible for sending out emails to all registered nurses working in their respective public health units, inviting them to voluntarily participate in the study. Invitation emails included a study introductory letter with a link to the written participant consent form and anonymous online survey.

All registered nurses within the participating health units were invited to enrol in the study. The target sample size was 400 participants based on a recommended 10:1 respondent to item ratio for factor analysis [42] and given the original EIDM competence tool consists of 40 items. Inclusion criteria were: 1) licensure as a registered nurse; 2) employment in an Ontario public health unit; and 3) working in any nursing or non-nursing role (e.g., health promoter) within the health unit. Those who participated in the first phase of the study (response process testing) were not eligible to participate, as they would have had familiarity with the measure, with potential to bias the results.

### Measures

Data collection occurred from December 2019 to March 2020. Upon confirming written consent via an online form, participants completed a one-time anonymous online survey via the LimeSurvey platform. The self-report survey consisted of three sections: 1) demographics; 2) organizational factors; and 3) the 40-item self-report EIDM Competence Measure (see S1 File). Demographic information collected included: number of years worked as a registered nurse and in public health; gender; current role; primary area of work specialization; highest earned degree; completion of formal EIDM training; and involvement in EIDM projects/work. Regarding organizational factors, 12 out of 19 items relevant to a public health setting, from the Organizational Culture and Readiness for System-Wide Implementation of EBP (OCR-SIEP) scale [43] were used with participants responding to items on a 5-point Likert scale (1 = not at all to 5 = very much). Since the OCR-SIEP employed a summative score, this same scoring process was applied to the subset of 12 selected items, with higher scores denoting greater organizational readiness for EIDM. Permission for use of scale items was granted by the original developer, Dr. Bernadette Melnyk [44]. The original 40-item self-report EIDM Competence Measure, which was assessed for content and response process validity in the first phase of the study [39], consisted of four subscales: knowledge (11 items), skills (10 items), attitudes/beliefs (7 items), and behaviours (12 items). Participants responded to all subscale items on a 7-point Likert scale: knowledge (1 = poor to 7 = excellent); skills (1 = beginner to 7 = expert); attitudes/beliefs (1 = strongly disagree to 7 = strongly agree); and behaviours (1 = not competent to 7 = highly competent). Given that the instrument was capturing different constructs, an overall total score was not computed. Instead, a total for each of the four subscales was computed. Reverse coding was conducted for only one item in the EIDM attitudes/beliefs subscale (i.e., I believe EIDM is difficult). Higher total subscale scores denoted higher competence for that EIDM competence attribute.

### Data analysis

#### Acceptability

Acceptability is associated with the practicality of a measure and was operationalized by the amount of time respondents were required to complete the tool and the extent to which there was difficulty with completion as measured by the amount of missing data [45]. Mean completion times were analyzed for the entire original 40-item tool and each EIDM subscale. Acceptable completion time was identified as < 10 minutes [46, 47] for the entire original 40-item tool. Missing data were analyzed through: 1) percentage of participants who completed the entire 40-item tool (i.e., no missing data) [48]; 2) percentage of participants who had data missing for at least one item [49]; and 3) percentage of participants who had >10% of missing data [50] across the entire 40-item tool (i.e., more than 4 out of 40 items with missing data).

#### Validity evidence based on internal structure

Validity evidence based on internal structure is defined as the “degree to which the relationships among test items and test components conform to the construct on which the proposed test score interpretations are based” [37 p. 36]. Internal structure was assessed by performing the following analyses using SAS 9.4 statistical software: 1) item-subscale total polychoric correlations, representing the correlation of individual items with subscale totals [41]; 2) item-item polychoric correlations within each subscale [51]; and 3) exploratory factor analysis using principal components analysis (PCA) using an oblique rotation which allowed for correlated factors, a common method used to extract potential latent variables/factors in the assessment of dimensionality and to reduce item components into more meaningful data [51–53]. Polychoric correlations were computed given that response scales of all items included in the tool consisted of ordinal data (i.e., 7-point Likert scales) [54, 55]. The use of polychoric correlations in the factor analysis of ordinal data, compared to Pearson correlations which are to be used with at least interval-level data, yields results that demonstrate less error and better alignment with originally proposed theoretical models [54].

Conceptual literature was considered alongside statistical criteria in decision-making related to item deletions or item combinations [56]. Items in which item-subscale correlations were low (<0.3) were deleted [41]. Item-item correlations within subscales were analyzed and those with low correlations <0.30 were flagged for possible deletion [41]. High item-item correlations within subscales of >0.80 may signal redundancy [51] and as such, were flagged for possible item deletion or item combination. Potential item deletions and merging of similar items were discussed and finalized with consideration of conceptual literature [51] among co-investigators given the team’s expertise in EIDM and public health nursing.

Following the item reduction process, a revised EIDM competence measure was proposed and used in conducting PCA. Prior to conducting PCA, the Kaiser-Meyer-Olkin (KMO) test was performed to determine sampling adequacy [51, 52] with a value above 0.5 being used to determine adequacy [42, 57]. PCA was performed using an oblique rotation, given that items were assumed to be interrelated [51]. A four factor model solution was forced in the PCA analysis, consistent with the design of the tool which was based on a priori conceptual literature [58] that EIDM competence is comprised of four key attributes of knowledge, skills, attitudes/beliefs, and behaviours. Items were assessed as loading onto a factor if loadings were a minimum of ≥ 0.4 [51], with strong loadings indicated by values of ≥ 0.5 [58, 59].

#### Validity evidence based on relations to other variables

Validity evidence based on variable relationships was assessed using the revised EIDM competence measure. This source of validity evidence involves the testing of relationships between instrument subscale scores and socio-demographic and organizational factors to determine their consistency with the construct under measurement [40]. A large body of evidence has shown that measurement scales created from at least four Likert-response format items are often linear and interval, unlike the ordinal scale applicable to the items in the scale [60]. This emergent property of measurement scales allows for parametric methods to be used, providing other key parametric assumptions are met. We tested the underlying assumptions of the parametric tests involved in the subscale analyses (i.e., normality, linearity, constant variance). A moderate departure from normality was seen for the skills and attitudes subscales (each comprised of five items), a minor departure for the knowledge subscale comprised of seven items, and no departure for the 10-item behaviour subscale. A linear relationship was seen between all four subscales and two continuous variables (years as a RN, organization tool score). The constant variance assumption held for all four subscales for two group variables (education, professional role). On the basis of these results, parametric tests were used in the sub-scale analyses. Correlations, t-tests, and ANOVA analyses were performed using IBM SPSS version 26 with level of significance set at alpha = 0.05 (2-sided) to explore variable relationships. The following relationships were hypothesized: 1) years of experience as a registered nurse would be positively correlated with EIDM knowledge, skills, attitudes, and behaviours [49]; 2) those working in a supervisory or management role would have higher EIDM knowledge, skills, attitudes/beliefs, behaviours compared to frontline staff [61, 62]; 3) those with a higher education level would have higher EIDM knowledge, skills, attitudes/beliefs, behaviour scores [61, 63]; 4) those who completed EIDM training or have had involvement in EIDM projects/work would have higher EIDM knowledge, skills, attitudes/beliefs, behaviour scores [63]; and 5) those who self-report higher organizational support for EIDM would have higher EIDM knowledge, skills, attitudes/beliefs, behaviour scores compared to those with lower organizational support [17, 19].

#### Reliability

Reliability was assessed by examining the measure’s internal consistency, which determines how well scale items are correlated with one another in order to yield similar scores [41, 64]. Cronbach’s alphas were computed for each individual subscale [41]. This is the most frequently used statistic for examining reliability in psychometric testing, can be determined with one administration, and is recommended for use with items that have more than two response options [41, 64]. Acceptable internal consistency for each sub-scale was determined as a Cronbach’s alpha (α) of ≥ 0.70 [65].

## Results

### Demographics

Sixteen Medical Officers of Health agreed to support the study, yielding a response rate of 50% (16/32). Across the 16 participating public health units, 562 registered nurses opened the online survey. Of these, 201 respondents (35.8%) completed and submitted the survey. Participants were largely female (98.5%), primarily employed as a frontline public health nurse (87.2%), Bachelor’s degree prepared (73.1%), and worked across diverse specializations, with the majority having completed EIDM training (66.8%). See Table 2 for detailed demographics.

**Table 2:** Participant demographics.

Demographic variable (N = 201)	
**Number of years worked**	**Mean (Standard Deviation)**
As a registered nurse (RN)	18.1 (10.7)
In public health	13.6 (8.5)
**Gender**	**n (%)**
Female	197 (98)
Male	2 (1)
Other	1 (0.5)
Missing	1 (0.5)
**Current Role**	
Public health nurse	171 (85)
Health promoter	3 (1.5)
Supervisory/manager	12 (6)
Director	2 (1)
Other	8 (4)
Missing	5 (2.5)
**Highest earned degree**	
Bachelor’s degree	147 (73.1)
Master’s degree	54 (26.9)
**Area of work specialization**	
Reproductive/infant health/healthy babies/children	61 (30.3)
School years	23 (11.4)
Chronic disease prevention/injury prevention and safety	16 (8)
Communicable diseases/sexually transmitted diseases	60 (29.8)
Emergency preparedness	3 (1.5)
Mental health	1 (0.5)
Substance use	13 (6.5)
Other	24 (11.9)
**Completed EIDM training (N = 199)**	
No	66 (32.8)
Yes	133 (66.2)
**Missing**	2 (1)
**Involvement in EIDM work/projects**	
No	102 (50.7)
Yes	99 (49.3)

### Acceptability

#### Completion time

The average completion times for each EIDM competence subscale were similar in length: knowledge (1 minute and 37 seconds); skills (2 minutes and 11 seconds); attitudes/beliefs (1 minute and 18 seconds); and behaviours (2 minutes and 14 seconds). The mean completion time for the entire original 40-item EIDM Competence Measure was 7 minutes and 20 seconds.

#### Missing data

The percentage of nurses fully completing the original 40-item EIDM measure (i.e., no missing data) was 93% (n = 187), with 7% of participants who had data missing for at least one item across the entire 40-item tool. As well, only one participant (0.5%) had >10% of missing data (i.e., more than 4 out of 40 items with data missing).

### Validity evidence

#### Validity based on internal structure

Item-subscale total correlations for the original 40 items all met the minimum criteria of >0.3 (see Table 3). Ranges of item-item correlations varied across subscales: knowledge (0.81–0.94); skills (0.81–0.92); attitudes/beliefs (0.04–0.87); and behaviours (0.80–0.90). Some item-item correlations fell below the minimum of 0.3 indicating weak relationships while others exceeded 0.8 indicating potential redundancy (see S1 Table for low and high item-item correlations). Based on low or high item-item correlations, along with consideration of conceptual literature, item deletions and item combinations were made within each EIDM subscale: 1) knowledge (deleted items K3, K4, K5, K7 based on redundancy); 2) skills (deleted items S2, S7, S8, S9 and combined items S3 and S4 based on redundancy); 3) attitudes (deleted item A7 due to irrelevance and combined items A4 and A6 due to redundancy); and 4) behaviours (deleted B5 and B6 due to redundancy).

**Table 3:** Item-subscale total correlations (40 items).

Item	Item-subscale correlation
**Knowledge**	
**K1. Knowledge of what is involved in the ’define’ step of EIDM**	**0.83**
**K2. Knowledge of what is involved in the ’search’ step of EIDM**	**0.86**
K3. Knowledge about different levels of evidence when searching for research evidence (e.g., single studies, systematic reviews, summaries)	0.87
K4. Knowledge that online databases exist which house publications of individual research studies (e.g., PubMed, CINAHL)	0.82
K5. Knowledge that online databases exist which house pre-appraised, synthesized research evidence (e.g., Health Evidence, ACCESSSS)	0.85
**K6. Knowledge of what is involved in the ’appraise’ step of EIDM**	**0.94**
K7. Knowledge that critical appraisal tools exist to assess the quality of research evidence (e.g., AGREE II tool, CASP)	0.85
**K8. Knowledge of what is involved in the ’synthesize’ step of EIDM**	0.94
**K9. Knowledge of what is involved in the ’adapt’ step of EIDM**	0.93
**K10. Knowledge of what is involved in the ’implement’ step of EIDM**	0.89
**K11. Knowledge of what is involved in the ’evaluate’ step of EIDM**	0.87
**Skills**	
**S1. Ability to develop an answerable practice question.**	**0.89**
S2. Ability to develop an appropriate strategy to search for research evidence	0.93
**S3. Ability to use online databases that house publications of individual research studies (e.g., CINAHL)**	0.85
**S4. Ability to use online databases that house pre-appraised, synthesized research evidence (e.g., Health Evidence)**	0.90
**S5. Ability to use critical appraisal tools to appraise the quality of research evidence (e.g., AGREE II tool, CASP)**	0.88
**S6. Ability to assess applicability of research evidence to the local public health context.**	0.94
S7. Ability to conduct an assessment of barriers and facilitators (related to resources, organization, evidence/guideline, clients’ preferences/values) when implementing a practice change.	0.92
S8. Ability to conduct a stakeholder analysis (i.e., collecting and analyzing information on stakeholders’ importance and influence) when implementing a practice change.	0.92
S9. Ability to develop an action plan to implement an evidence-informed practice change.	0.92
**S10. Ability to participate in the development of evaluation indicators to assess outcomes of evidence-informed decision or practice changes.**	0.91
**Attitudes**	
**A1. I believe that I can implement EIDM in a time efficient way.**	0.78
**A2. I believe I can engage others in implementing strategies to address barriers (e.g., personal, organizational, community) when implementing EIDM**	0.75
**A3. I believe that evaluating outcomes of an evidence-informed decision or practice change is an important component of EIDM.**	0.83
**A4. I believe that implementing EIDM can improve the services and programs delivered to clients (e.g., communities, individuals, families).**	0.83
**A5. I believe that critically appraising evidence is an important step in the EIDM process.**	0.77
**A6. I believe that the use of high-quality evidence-informed guidelines (e.g., clinical practice guidelines) can improve public health practice and policy.**	0.73
A7. I believe EIDM is difficult.	0.52
**Behaviours**	
**B1. I question public health practices for the purpose of improving the quality of care/service delivery.**	0.69
**B2. I describe public health practice issues using client assessment data (i.e., community, individuals, families, populations).**	0.73
**B3. I participate in the formulation of public health practice questions.**	0.84
**B4. I search for research evidence to answer public health practice questions.**	0.70
B5. I participate in the critical appraisal of individual research studies to determine their strength and applicability to public health practice.	0.87
B6. I participate in the critical appraisal of synthesized evidence (such as clinical practice guidelines, evidence-based policies and procedures, and evidence syntheses).	0.84
**B7. I participate in the synthesis and interpretation of a body of research evidence gathered to formulate recommendations for public health practice.**	0.90
**B8. I integrate evidence gathered from public health expertise, client or community preferences, and local context with research evidence to plan evidence-informed practice changes.**	0.90
**B9. I participate in the assessment of barriers and facilitators (related to resources, organization, evidence/guidelines, clients’ preferences/values) when implementing a practice change.**	0.84
**B10. I participate in the process of stakeholder analyses (i.e., collecting and analyzing information on stakeholders’ importance and influence) when implementing a practice change.**	0.87
**B11. I participate in the development of an action plan to implement a practice change.**	0.84
**B12. I participate in evaluating outcomes of evidence-informed decisions or practice changes.**	0.85

Note: Bolded items represent the remaining 27-items in the final PCA. “Skills” items #3 and #4 were combined and “Attitudes” items #4 and #6 were combined for the final PCA.

Following the item reduction process, a revised EIDM competence measure was proposed consisting of 27 items.

A PCA with oblique rotation was then performed on the revised 27-item self-report Competence Measure. The Kaiser-Meyer-Olkin test verified sampling adequacy with a value of 0.8597. A four-factor model was extracted with all factors accounting for 90.00% of the variance. Primary loadings were substantial across factors and ranged from 0.46 to 0.92. Items primarily loaded onto factors that aligned with the established conceptual framework of EIDM behaviours (Factor 1), knowledge (Factor 2), skills (Factor 3), and attitudes/beliefs (Factor 4). However, there were three items which loaded onto factors to which they were not conceptually assigned: 1) attitude item #1 (believe can implement EIDM efficiently) loaded onto Factor 3 (skills, factor loading = 0.54); 2) attitude item #2 (believe can engage others to address EIDM barriers) loaded onto Factor #1 (behaviours, factor loading = 0.46); and 3) skills item #5 (ability to develop evaluation indicators) loaded onto Factor #1 (behaviours) with a value of 0.58 (see Table 4 for factor loadings). After reviewing these factor loadings, along with consideration of acceptable item-subscale total correlations (see Table 5) and conceptual literature, these three items were retained in the competence attribute under which they were originally categorized. The final proposed 27-item EIDM competence measure consisted of a varied number of items per subscale: knowledge (7 items); skills (5 items); attitudes (5 items); behaviours (10 items).

**Table 4:** Factor loadings for 27-item EIDM competence measure.

	Behaviours	Knowledge	Skills	Attitudes
B9 participate in development of action plan	**0.83**	0.36	0.06	0.06
B7 participate in assessment of barriers/facilitators	**0.83**	0.34	0.15	0.07
B10 participate in evaluating outcomes	**0.83**	0.35	0.11	0.08
B8 participate in stakeholder analysis	**0.80**	0.30	0.24	0.01
B6 integrate evidence from expert/preferences/context	**0.79**	0.29	0.30	0.11
B3 participate in formulating public health practice questions	**0.67**	0.23	0.40	0.16
B5 participate in synthesis and interpretation of evidence	**0.64**	0.28	0.49	0.09
B2 describe public health practice issues using client data	**0.60**	0.18	0.34	0.24
S5 ability to develop evaluation indicators	**0.58**	0.44	0.50	0.08
B1 question public health practices	**0.56**	0.13	0.35	0.24
B4 search for research evidence	**0.53**	0.04	0.36	0.40
A2 engage others to address EIDM barriers	**0.46**	0.34	0.43	0.25
K9 knowledge of adapt step	0.31	**0.83**	0.30	0.11
K6 knowledge of implement step	0.41	**0.82**	0.16	0.14
K4 knowledge of synthesize step	0.24	**0.81**	0.41	0.13
K1 knowledge of define step	0.25	**0.77**	0.18	0.18
K3 knowledge of appraise step	0.23	**0.76**	0.41	0.19
K7 knowledge of evaluate step	0.42	**0.74**	0.22	0.22
K2 knowledge of search step	0.33	**0.70**	0.28	0.26
S2 ability to use online databases	0.24	0.49	**0.70**	0.09
S3 ability to use critical appraisal tools	0.32	0.43	**0.69**	0.02
S1 ability to develop answerable public health question	0.37	0.50	**0.64**	0.07
S4 ability to assess applicability of research to local context	0.49	0.46	**0.63**	0.05
A1 believe can implement EIDM efficiently	0.40	0.43	**0.54**	0.26
A4 implementing EIDM improves services, programs, policies	0.08	0.12	0.06	**0.92**
A3 believe evaluation important	0.10	0.19	0.07	**0.86**
A5 believe critical appraisal is important	0.13	0.19	0.06	**0.84**

**Note:** A = attitude item; B = behaviour item; K = knowledge item; S = skills item

**Table 5:** Item-subscale total correlations for 27-item EIDM competence measure.

Item	Item-subscale correlation
**Knowledge**	
1. Knowledge of what is involved in the ’define’ step of EIDM	0.86
2. Knowledge of what is involved in the ’search’ step of EIDM	0.88
3. Knowledge of what is involved in the ’appraise’ step of EIDM	0.92
4. Knowledge of what is involved in the ’synthesize’ step of EIDM	0.95
5. Knowledge of what is involved in the ’adapt’ step of EIDM	0.96
6. Knowledge of what is involved in the ’implement’ step of EIDM	0.94
7. Knowledge of what is involved in the ’evaluate’ step of EIDM	0.91
**Skills**	
1. Ability to develop an answerable practice question.	0.92
2. Ability to use online databases that house research evidence (combined original skills item #3 and #4).	0.90
3. Ability to use critical appraisal tools to appraise the quality of research evidence (e.g., AGREE II tool, CASP)	0.91
4. Ability to assess applicability of research evidence to the local public health context.	0.94
5. Ability to participate in the development of evaluation indicators to assess outcomes of evidence-informed decision or practice changes.	0.90
**Attitudes**	
1. I believe that I can implement EIDM in a time efficient way.	0.84
2. I believe I can engage others in implementing strategies to address barriers (e.g., personal, organizational, community) when implementing EIDM	0.81
3. I believe that evaluating outcomes of an evidence-informed decision or practice change is an important component of EIDM.	0.84
4. I believe that implementing EIDM can improve public health services, programs, and policies (combined original attitudes items #4 and #6).	0.78
5. I believe that critically appraising evidence is an important step in the EIDM process.	0.79
**Behaviours**	
1. I question public health practices for the purpose of improving the quality of care/service delivery.	0.71
2. I describe public health practice issues using client assessment data (i.e., community, individuals, families, populations).	0.75
3. I participate in the formulation of public health practice questions.	0.85
4. I search for research evidence to answer public health practice questions.	0.70
5. I participate in the synthesis and interpretation of a body of research evidence gathered to formulate recommendations for public health practice.	0.86
6. I integrate evidence gathered from public health expertise, client or community preferences, and local context with research evidence to plan evidence-informed practice changes.	0.91
7. I participate in the assessment of barriers and facilitators (related to resources, organization, evidence/guidelines, clients’ preferences/values) when implementing a practice change.	0.90
8. I participate in the process of stakeholder analyses (i.e., collecting and analyzing information on stakeholders’ importance and influence) when implementing a practice change.	0.89
9. I participate in the development of an action plan to implement a practice change.	0.87
10. I participate in evaluating outcomes of evidence-informed decisions or practice changes.	0.88

#### Validity based on relationships to other variables

While some non-significant relationships between EIDM competence attributes and other variables were revealed, there were also statistically significant findings that confirmed many of the hypothesized relationships to establish validity evidence of the EIDM competence measure.

Regarding number of years worked as a registered nurse, a statistically significant positive correlation was found with EIDM behaviours, indicating a weak relationship (r = 0.17; *p* = 0.008), although no significant relationships were found related to EIDM knowledge, skills, and attitudes (see Table 6). Similarly, statistically significant differences in mean scores were found only for EIDM behaviours between professional role groups of public health nurse (M = 42.69; SD = 11.79), health promoter (M = 47.00; SD = 7.00), supervisor/manager (M = 49.75; SD = 8.87), director (M = 53.50; SD = 13.44), and other (M = 53.57; SD = 12.63); F(4, 187) = 2.80; *p* = 0.027 (see Table 7). However, a follow-up post-hoc Tukey’s test was performed for EIDM behaviour scores to attempt to pinpoint specific group differences, and no statistically significant relationships were identified.

**Table 6:** Correlation between EIDM subscale totals and years worked as RN.

Subscale	Mean (Standard Deviation)	Pearson correlation coefficient (r)	*p*
EIDM knowledge	31.20(9.14)	0.00	0.499
EIDM skills	20.11(7.19)	0.07	0.357
EIDM attitudes/beliefs	27.08(4.34)	-0.04	0.623
EIDM behaviours	43.99(11.97)	0.17	0.008

**Table 7:** One-Way ANOVA of EIDM subscale scores as a function of professional role.

Subscale	F	*p*
EIDM knowledge	1.08	0.369
EIDM skills	2.25	0.065
EIDM attitudes	0.88	0.477
EIDM behaviours	2.80	0.027

Higher scores in EIDM knowledge, skills, attitudes/beliefs, and behaviours were found among nurses with master’s degree preparation compared to those with a bachelor’s degree (*p*<0.0001; see Table 8 for mean scores). Differences between education groups were statistically significant for EIDM knowledge *t*(194) = 4.80, (*p*<0.0001); EIDM skills *t*(196) = 6.33, (*p*<0.0001); EIDM attitudes *t*(197) = 4.53, (*p*<0.0001); and EIDM behaviours *t*(195) = 5.50, (*p*<0.0001).

**Table 8:** Mean EIDM competence scores based on education.

Subscale	Education Level	N	Mean	Standard Deviation
EIDM Knowledge	bachelor’s degree	143	29.39	8.49
	master’s degree	53	36.08	9.13
EIDM Skills	bachelor’s degree	145	18.33	6.61
	master’s degree	53	25.00	6.43
EIDM Attitudes	bachelor’s degree	145	26.27	4.31
	master’s degree	54	29.26	3.61
EIDM Behaviours	bachelor’s degree	144	41.33	11.33
	master’s degree	53	51.21	10.71

Higher scores across were also found among nurses who completed training in EIDM compared to those who did not across EIDM knowledge *t*(192) = 6.49, *p*<0.0001; EIDM skills *t*(194) = 5.5, (*p*<0.0001); EIDM attitudes *t*(195) = 3.69, (*p*<0.0001); and EIDM behaviours *t*(193) = 4.86, (*p*<0.0001). Compared to those with no EIDM experience, participants with involvement in EIDM related work or projects had higher scores in EIDM knowledge *t*(188.03) = 6.06, *p*<0.0001; EIDM skills *t*(191.59) = 6.91, (*p*<0.0001); EIDM attitudes *t*(197) = 3.69, (*p*<0.0001); and EIDM behaviours *t*(195) = 6.72, (*p*<0.0001). Statistically significant positive correlations were found between total organizational factor scores and EIDM knowledge (r = 0.29; *p*<0.000), skills (r = 0.27; p = 0.001), attitudes/beliefs (r = 0.26; *p* = 0.00), and behaviours (r = 0.22; *p* = 0.005).

In summary, statistically significant positive relationships were found between EIDM behaviours and numbers of years worked as a registered nurse, as well as professional role group. Higher scores across all four subscales were also found among nurses with higher education levels and among those who completed EIDM training and had EIDM work experience. Positive correlations were also found between higher organizational support scores and EIDM subscale scores.

### Internal consistency reliability

All EIDM subscales from the 27-item tool met the minimum Cronbach’s alpha (α) of ≥0.70: knowledge (α = 0.96); skills (α = 0.93); attitudes/beliefs (α = 0.80); and behaviours (α = 0.94).

## Discussion

This study reports on the acceptability, reliability, and validity of the new EIDM Competence Measure which assesses EIDM knowledge, skills, attitudes, and behaviours among public health nurses. Generally, existing EIDM measures developed, tested, and used among diverse groups of nurses have focused primarily on the assessment of a single competence attribute [35]. As well, limited attention has been given to EIDM competence assessment of nurses working in public health, compared to other nursing practice areas such as acute care [35]. Of note, only one existing measure, the School-Nursing Evidence Based Practice Questionnaire [49] assesses all four competence attributes of knowledge, skills, attitudes, and behaviours and has been tested among a sample of public health nurses in the United States. However, the measure has conceptual limitations in that it does not measure the quality or competence of these attributes but focuses only on frequency of use. As well, items are designed specifically for the nuanced field of school health nursing, with little applicability to other nursing practice areas in public health. The newly developed EIDM Competence Measure contributes to existing gaps within the EIDM competence literature and has applicability for use across all fields of public health nursing to assess their EIDM competence.

### Acceptability

Minimal missing data and the short completion time observed in this study suggest that our EIDM Competence Measure is ‘acceptable’, signifying it is not highly burdensome or challenging to complete [66]. The overall low percentage of missing data for the EIDM competence measure (7%) resembles rates of other EIDM measures that have been used or tested among public health nurses including the EBP Implementation Scale (6.3%) [67] and the School Nurse Evidence-Based Practice Questionnaire (5.2%) [49]. As well, the original 40-item EIDM Competence Measure appears to have a similar completion time to other EIDM measures of shorter length which assess only one competence attribute: 16-item EBP Beliefs Scale (~5–7 minutes) [68, 69]; 18-item EBP Implementation Scale (~6–8 minutes) [68, 69].

### Validity evidence

Findings from this study provide beginning validity evidence related to internal structure and relationships to other variables of the self-report EIDM Competence Measure for public health nursing. Principal components analysis results for the EIDM competence measure supported a four-factor model that aligned with the conceptual understanding that EIDM competence is comprised of four attributes of knowledge, skills, attitudes/beliefs, and behaviours. In comparison, results from the psychometric assessment of another self-report measure [36] addressing these same competence attributes yielded to some extent, different factor compositions. Principal component analysis of the 24-item Evidence Based Practice Questionnaire (EBPQ) produced a three-factor model for the measure: practice of evidence-based practice (related to behaviour frequency); attitudes towards evidence-based practice; and knowledge/skills associated with evidence-based practice [36]. EIDM behaviours and attitudes emerged as separate entities in the EBPQ, a similar finding in our EIDM competence measure. As well, in the EBPQ, knowledge and skills items appeared to be highly related, loading together to comprise one factor [36]. In the analysis of our self-report EIDM Competence Measure, knowledge emerged as a distinct factor independent of others. This difference in terms of knowledge surfacing as a distinct factor may have been attributed to the broad nature in which knowledge items were articulated compared to the specificity used to formulate items in the other subscales of skills, attitudes, and behaviours in the EIDM Competence Measure. While, in the EBPQ, both the knowledge and skills items were worded and phrased similarly, possibly contributing to their emergence as one factor. In our study, there were also two instances in which attitude items (i.e., Beliefs in implementing EIDM efficiently and Engaging others to address EIDM barriers) loaded onto factors representing EIDM behaviour or skills. Looking at the phrasing of these two attitude items, since they relate to beliefs/perceptions about personal engagement in EIDM overall and in ability to address EIDM barriers, it seems reasonable that statistically, they might cluster together with items that are phrased similarly to assess ability, participation or performance of EIDM tasks. However, regardless of factor loadings, conceptually, these items are better represented under the ‘attitudes’ attribute given these are defined as the values, perceptions, beliefs or intentions related to EIDM. This may include acceptance of, motivation or self-efficacy in overall EIDM engagement [30, 33], as compared to categorization under EIDM skills (application of knowledge to perform discrete EIDM tasks in a practical setting) [30, 33, 34] or EIDM behaviours (EIDM performance in real-world practice) [30, 33, 34].

Results in this study also showed evidence to support validity based on relationships with other variables for the EIDM competence measures. In our study, there were statistically significant associations between all EIDM competence attributes and education level, EIDM training, and EIDM work experience. These findings are consistent with other literature indicating that EIDM engagement is heavily influenced by personal and professional characteristics such as having advanced level formal education [63], exposure to multifaceted EIDM educational interventions [70, 71], and opportunities to participate in or lead EIDM projects in real-world practice [63, 72]. Our study findings also align with existing literature that consistently demonstrates organizational context as a strong predictor of EIDM uptake. Similar to other studies, EIDM knowledge, skills, attitudes/beliefs and behaviours were all significantly related to work environments in which EIDM priorities were integrated into strategic plans [17, 73], there were identified EIDM champions [74, 75], and critical resources necessary to carrying out EIDM activities were provided [76, 77].

There were however some findings in which the relationships we hypothesized were not validated. Our findings did not show a significant positive correlation between years of experience as a registered nurse and EIDM competence attributes. While many studies have determined that a longer duration of work experience is associated with more developed EIDM competence attributes [78–80], literature has emerged showing conflicting evidence; that there is either no existing relationship between these variables [61] or that those with less nursing experience actually display higher EIDM competence attribute scores [81]. Regarding the influence of professional role, our study findings demonstrated only a significant relationship between role (e.g., public health nurse, supervisor/manager) and EIDM behaviours specifically, but this relationship was not found regarding knowledge, skills, or attitudes. This is in contrast to other studies with reported findings that showed higher level professional roles (e.g., management, advanced practice nurse) were associated with greater scores on EIDM knowledge, skills, attitudes, and behaviours [77, 81, 82]. However, an important note is that these existing studies pertain to an acute care setting where discussions of advanced roles were focused on clinical distinctions (i.e., frontline staff nurse versus educator or nurse practitioner). In comparison, our study, set in a public health context in which these clinical roles are less prominent, explored roles in relation to public health nurses, health promoters, and management, which may have contributed to differences in findings.

This study’s findings add to previously established validity evidence related to test content and response process [39] for the EIDM Competence Measure. Based on the Standards for Educational and Psychological Testing, which suggests that validity is a unified concept consisting of four types of validity evidence (content, internal structure, response process, relationships to other variables), study findings provide cumulative validity evidence for the EIDM Competence Measure.

### Reliability

The EIDM Competence Measure also exhibited strong internal reliability with Cronbach’s alphas for all subscales exceeding the minimum of 0.70 for new measures [65]. Given these high alphas, which may be indicative of further redundancy [41], there is opportunity for possible refinement of the tool in further psychometric testing with other populations and contexts. Similarly, original psychometric testing of the EBPQ also yielded high alphas for the EBP practice (behaviour) subscale (α = 0.85) and the combined knowledge/skills subscale (α = 0.91). Assessment of the Quick Evidence-Based Practice (EBP) Values, Implementation and Knowledge (VIK) survey, a 25-item self-report tool addressing EIDM knowledge, attitudes/beliefs, and behaviours, also demonstrated comparable internal consistency with its subscale of knowledge (α = 0.93), although had a lower alpha (0.76) for its implementation subscale (frequency of EIDM behaviours) [83]. This latter discrepancy may be attributed to item content differences in which behavioural items of the Quick-EBP-VIK survey are less specific and have less coverage of all the EIDM steps compared to our EIDM competence measure or the EBPQ. What remains consistent across the psychometric literature are reported alpha values for EIDM attitude/beliefs subscales across multi-dimensional measures: our EIDM competence measure (α = 0.80); EBPQ (α = 0.79) [36]; and the Quick-EBP-VIK survey (α = 0.79) [83].

### Limitations

While this study provides supporting evidence of the acceptability, validity, and reliability of a new EIDM competence measure that can be used in public health practice, there are limitations that require consideration. While the proposed study sample size was 400, only 50% (n = 201) of this projected sample was achieved. As such, given the original EIDM competence measure had 40 items, the frequently recommended ratio of 10:1 (subjects to items) in calculating sample size for factor analysis was not met [51]. Given that the principal components analysis was instead run on the reduced 27-item tool, using the 10:1 subject to items ratio, an acceptable sample size would be 270, which still was not met with the sample size of 201 participants. However, the Kaiser-Meyer-Olkin test determined that there was acceptable sampling adequacy to conduct factor analysis in this study. As well, in other literature, a case to variable ratio of 5:1 has also been deemed sufficient to conduct factor analysis [84]. Comrey as cited in Taherdoost et al. [57] identifies 200 as a ‘fair’ sample, compared to increasing samples of 300 classified as ‘good’ and 500 respondents as ‘very good’. As well, given the high non-response rate and the use of convenience sampling, this presents non-response and self-selection bias, impacting the representativeness of the sample. The sample of public health nurses surveyed may not represent the true diversity among the public health nursing population employed throughout Ontario, influencing the generalizability of results. However, given challenges with accessing public health nurse employee lists across Ontario health units to support random sampling, convenience sampling was determined as the most feasible choice. It is important to note that this study is considered exploratory in nature, with an understanding that findings are to be interpreted with caution. A next step will be to use a larger national study sample to conduct confirmatory factor analysis with a split sample approach, which was not feasible to perform in this pilot study given the sample size [85].

Over the course of this study, particularly throughout the study recruitment period, there were pivotal public health events that had substantial bearing on the ability to recruit the proposed sample size. First, the provincial government of Ontario announced public health modernization plans which proposed a critical restructuring of the public health system, reducing the number of operating health units in Ontario from 36 to 10. Second, the beginning stages of the COVID-19 coronavirus pandemic had emerged during this time, with health units dedicating staff resources toward the pandemic response. These two events impacted study participation at both the public health unit and staff level and illustrates some of the challenges of conducting research in a health sector that regularly responds to emerging crises. The changing nature of public health illuminates the need to embed strategies that will mitigate the impact of unforeseen and unavoidable circumstances within the research design.

## Conclusions

The 27-item EIDM competence measure provides a comprehensive self-assessment of EIDM knowledge, skills, attitudes/beliefs, and behaviours for use among nurses in public health practice. This instrument has demonstrated beginning validity evidence based on internal structure and relations to other variables, as well as exhibits strong internal reliability. Given its ease of use and short completion time, there is great potential for its use in real-world public health practice for individual nurses, supervisors/managers, and organizations to provide insight into the status of EIDM competence among public health nurses. This sets the stage well for improving clarity around EIDM expectations for nursing and potentially other public health professions and in strategic planning of resources and professional development interventions to facilitate improved EIDM engagement. Given the nature of this study as a pilot, there is opportunity to expand on the psychometric testing conducted which would include confirmatory factor analysis/split sample approach using a national sample of nurses across health units in Canada, continuing to add to the reliability and validity evidence of the EIDM Competence Measure, as well as explore its acceptability across a diverse sample.

## Supporting information

S1 File Participant survey. (DOCX)

S1 Table Low and high item-item correlations (40 items). (DOCX)
