# Supplementary material for: Validity, reliability, and acceptability of the Evidence-Informed Decision-Making (EIDM) competence measure
Source: PLoS One. 2022 Aug 5;17(8):e0272699. doi: 10.1371/journal.pone.0272699 (PMC9355195; doi:10.1371/journal.pone.0272699)
Supplement: S1 File — (DOCX) [file pone.0272699.s001.docx]

**S1 File. Participant survey**

Registered nurses from public health departments throughout Ontario are being invited

to complete this survey as part of the study “Measuring Evidence-Informed Decision-Making Competence in Public Health Nursing”.

Please complete this survey if you are a registered nurse working in any role across any division in your health unit.

The survey will take approximately 20 minutes to complete. Your responses to the survey are anonymous. You will not be identified in the reporting of the results.

**Demographics**

1. Number of years worked as a Registered Nurse:
2. Number of years worked in Public Health:
3. Role:
   - Frontline Public Health Nurse
   - Health Promoter
   - Policy Analyst
   - Supervisor/Manager
   - Director
   - Other. Please specify:
4. Gender
   - Male
   - Female
   - Other
5. Area of work specialization. Choose only ONE/PRIMARY area of work.
   - Reproductive/infant health
   - Healthy Babies/Healthy Children (HBHC)
   - Pre-school
   - School years
   - Chronic disease prevention (e.g., nutrition, physical activity)
   - Communicable and infectious diseases (e.g., rabies, tuberculosis, vaccine preventable diseases)
   - Dental/oral health
   - Emergency preparedness
   - Environmental health (e.g., food and water safety, health hazard prevention)
   - Injury prevention/safety
   - Mental health
   - Sexual health / sexually transmitted infections-STIs
   - Social determinants of health
   - Substance use / misuse / addiction
   - Other. Please specify ONE/PRIMARY area of specialization:
6. Highest earned degree in Nursing:
   - Diploma
   - Bachelor’s degree
   - Master’s degree
   - Doctoral degree
7. Have you completed training in evidence-based practice (EBP)/evidence-informed decision making (EIDM) (e.g., webinar, in-person course):
   - No
   - Yes
8. Have you ever (currently or in the past) been involved in any EIDM-related activities/projects at work (e.g., participating/leading a rapid review, training other individuals in EIDM, participation in a journal club):
   - No
   - Yes
9. Have you been involved in program planning?
   - No
   - Yes
     - If yes, which one:
       - Pilot
       - Cohort 1 and 2

**Organizational Factors**

*Reference:* Fineout-Overholt E, Melnyk BM. Organizational Culture and Readiness for System Wide Implementation of EBP (OCRSIEP) Scale. ARCC llc Publishing; Gilbert, AZ: 2006.

| Item | None at all | A Little | Somewhat | Moderately | Very Much |
| --- | --- | --- | --- | --- | --- |
| 1. To what extent is EIDM clearly described as central to the mission and philosophy of your institution? | 1 | 2 | 3 | 4 | 5 |

| 2. To what extent is the nursing staff  with whom you work committed to EIDM? | 1 | 2 | 3 | 4 | 5 |
| --- | --- | --- | --- | --- | --- |
| 3. To what extent are there administrators within your organization committed to EIDM (i.e., have planned resources and support [e.g., time] to initiate EIDM)? | 1 | 2 | 3 | 4 | 5 |
| 4. In your organization, to what extent is there a critical mass of nurses who have strong EIDM knowledge and skills? | 1 | 2 | 3 | 4 | 5 |
| 5. To what extent do practitioners model EIDM in their clinical setting? | 1 | 2 | 3 | 4 | 5 |
| 6. To what extent do staff nurses have access to quality computers and access to electronic databases for searching for best evidence? | 1 | 2 | 3 | 4 | 5 |
| 7. To what extent are fiscal resources used to support EIDM (e.g. education-attending EIDM conferences/workshops, computers, paid time for the EIDM process, mentors) | 1 | 2 | 3 | 4 | 5 |
| 1. To what extent are there EIDM champions (i.e., those who will go the extra mile to advance EIDM) in the environment among:    - Administrators    - Physicians    - Nurse Educators    - Advance Nurse Practitioners    - Staff Nurses | 1 | 2 | 3 | 4 | 5 |

**EIDM Competence Attributes: Knowledge, Skills, Attitudes/Beliefs, Behaviours**

In the following questions, you will be asked to assess your own knowledge, skills, attitudes/beliefs and behaviours in EIDM.

**Definitions:**

**Evidence-informed decision-making (EIDM)**: “the process of distilling and disseminating the best available evidence from research, context and experience, and using that evidence to inform and improve public health practice and policy” (The National Collaborating Centre for Methods and Tools, 2018). According to the National Collaborating Centre for Methods and Tools (2018), there are seven steps that define EIDM:

1. **Define:** Clearly define the question or problem.
2. **Search:** Efficiently search for research evidence.
3. **Appraise:** Critically and efficiently appraise the research sources.
4. **Synthesize**: Interpret/form recommendations for practice based on the literature found.
5. **Adapt:** Adapt the information to a local context.
6. **Implement:** Decide whether (and plan how) to implement the adapted evidence into practice or policy.
7. **Evaluate:** Evaluate the effectiveness of implementation efforts.

**Competence**: “the ability to perform the tasks and roles required to the expected standard” (Eraut, 1998, p. 129) in a specific domain. It encompasses four attributes of knowledge, skills, attitudes/values, and behaviours (Cheetham & Chivers, 1998).

**EIDM Knowledge:** Understanding the defining theoretical, practical concepts and principles of EIDM and the different levels of evidence

Please rate your level of knowledge for each of the items on a scale from **(1) Poor to (7) Excellent**

| 1. Knowledge of what is involved in the ‘define’ step of EIDM. | 1 2 3 4 5 6 7    Poor Excellent |
| --- | --- |
| 2. Knowledge of what is involved in the ‘search’ step of EIDM. | 1 2 3 4 5 6 7    Poor Excellent |
| 3. Knowledge about the different levels of evidence when searching for research evidence (e.g., single studies, systematic reviews, summaries) | 1 2 3 4 5 6 7    Poor Excellent |
| 4. Knowledge that online databases exist which house publications of individual research studies (e.g., PubMed, CINAHL) | 1 2 3 4 5 6 7    Poor Excellent |
| 5. Knowledge that online databases exist which house pre-appraised, | 1 2 3 4 5 6 7 |

| synthesized research evidence (e.g.,  Health Evidence, ACCESSSS) | -         Poor Excellent |
| --- | --- |
| 6. Knowledge of what is involved in the ‘appraise’ step of EIDM. | 1 2 3 4 5 6 7    Poor Excellent |
| 7. Knowledge that critical appraisal tools exist to assess the quality of research evidence (e.g., AGREE II tool, CASP). | 1 2 3 4 5 6 7    Poor Excellent |
| 8. Knowledge of what is involved in the ‘synthesize’ step of EIDM. | 1 2 3 4 5 6 7    Poor Excellent |
| 9. Knowledge of what is involved in the ‘adapt’ step of EIDM. | 1 2 3 4 5 6 7    Poor Excellent |
| 10. Knowledge of what is involved in the ‘implement’ step of EIDM. | 1 2 3 4 5 6 7    Poor Excellent |
| 11. Knowledge of what is involved in the ‘evaluate’ step of EIDM. | 1 2 3 4 5 6 7    Poor Excellent |

**EIDM Skills:** The application of EIDM knowledge to perform tasks related to EIDM in a practical setting

Please rate your level of skill for each of the items from **(1) Beginner to (7) Expert**

| 1. Ability to develop an answerable practice question. | 1 2 3 4 5 6 7    Beginner Expert |
| --- | --- |
| 1. Ability to develop an appropriate strategy to search for research evidence. | 1 2 3 4 5 6 7    Beginner Expert |
| 1. Ability to use online databases that house publications of individual research studies (e.g., CINAHL) | 1 2 3 4 5 6 7    Beginner Expert |
| 1. Ability to use online databases that house pre-appraised, synthesized research evidence (e.g., Health Evidence). | 1 2 3 4 5 6 7    Beginner Expert |
| 1. Ability to use critical appraisal tools to appraise the quality of research evidence (e.g., AGREE II tool, CASP) | 1 2 3 4 5 6 7    Beginner Expert |
| 1. Ability to assess the applicability of research evidence to the local public health context. | 1 2 3 4 5 6 7    Beginner Expert |
| 1. Ability to conduct an assessment of barriers and facilitators (related to resources, organization, evidence/guideline, clients’ preferences/values) when   implementing a practice change. | 1 2 3 4 5 6 7    Beginner Expert |
| 1. Ability to conduct a stakeholder analysis (i.e. collecting and analyzing information on stakeholders’ importance and   influence) when implementing a practice change. | 1 2 3 4 5 6 7    Beginner Expert |
| 1. Ability to develop an action plan to implement an evidence-informed practice change. | 1 2 3 4 5 6 7    Beginner Expert |
| 1. Ability to participate in the development of evaluation indicators to assess outcomes of evidence-informed decisions or practice changes. | 1 2 3 4 5 6 7    Beginner Expert |

**EIDM Attitudes/Beliefs:** Perceptions, personal beliefs about, and the importance assigned to EIDM

*Reference:* Melnyk, B. M., Fineout-Overholt, E., & Mays, M. Z. (2008). The evidence-based practice beliefs and implementation scales: Psychometric properties of two new instruments. *Worldviews on Evidence-Based Nursing, 5*(4), 208-216. doi[:10.1111/j.1741-6787.2008.00126.x](https://dx.doi.org/10.1111/j.1741-6787.2008.00126.x)

Please rate your level of agreement with the following items **Strongly disagree (1) to Strongly agree (7)**.

| 1. 17. I believe that I can implement EIDM in a time efficient way. | 1 2 3 4 5 6 7    Strongly disagree Strongly agree |
| --- | --- |
| 1. 18. I believe that I can engage others in implementing strategies to address barriers (e.g., personal, organizational, community) when implementing EIDM. | 1 2 3 4 5 6 7    Strongly disagree Strongly agree |

| 1. 19. I believe that evaluating outcomes of an evidence-informed decision/practice change is an important component of EIDM. | 1 2 3 4 5 6 7    Strongly disagree Strongly agree |
| --- | --- |
| 1. 20. I believe that implementing EIDM can improve the services and programs delivered to clients (e.g., communities, individuals, families). | 1 2 3 4 5 6 7    Strongly disagree Strongly agree |
| 1. 21. I believe that critically appraising evidence is an important step in the EIDM process. | 1 2 3 4 5 6 7    Strongly disagree Strongly agree |
| 1. 22. I believe that the use of high- quality evidence-informed guidelines (e.g., clinical practice guidelines) can improve public health practice and policy. | 1 2 3 4 5 6 7    Strongly disagree Strongly agree |
| 1. 23. I believe EIDM is difficult. (reverse scored) | 1 2 3 4 5 6 7    Strongly disagree Strongly agree |

**EIDM Behaviours:** The enactment of EIDM steps in a real-life health care setting

*Reference:* Melnyk, B. M., Gallagher-Ford, L., Zellefrow, C., Tucker, S., Thomas, B., Sinnott, L. T., & Tan, A. (2018). The first U.S. study on nurses' evidence-based practice competencies indicates major deficits that threaten healthcare quality, safety, and patient outcomes. *Worldviews on Evidence-Based Nursing, 15*(1), 16-25.

**Please** rate your level of competence for the following items from **(1) Not competent to (4) Highly competent**

| 1. I question public health practices for the purpose of improving the quality of care/service delivery. | 1 2 3 4 5 6 7    Not competent Highly competent |
| --- | --- |
| 2. I describe public health practice issues using client assessment  data (i.e., community, individuals, families, populations). | 1 2 3 4 5 6 7    Not competent Highly competent |

| 3. I participate in the formulation of public health practice questions. | 1 2 3 4 5 6 7    Not competent Highly competent |
| --- | --- |
| 4. I search for research evidence to answer public health practice questions. | 1 2 3 4 5 6 7    Not competent Highly competent |
| 5. I participate in the critical appraisal of individual research studies to determine their strength and applicability to public health practice. | 1 2 3 4 5 6 7    Not competent Highly competent |
| 6. I participate in the critical appraisal of synthesized evidence (such as clinical practice guidelines, evidence-based policies and procedures, and evidence syntheses). | 1 2 3 4 5 6 7    Not competent Highly competent |
| 7. I participate in the synthesis and interpretation of a body of research evidence gathered to  formulate recommendations for public health practice. | 1 2 3 4 5 6 7    Not competent Highly competent |
| 8. I integrate evidence gathered from public health expertise, client/community preferences, and local context with research evidence to plan evidence- informed practice changes. | 1 2 3 4 5 6 7    Not competent Highly competent |
| 9. I participate in the assessment of barriers and facilitators (related to resources, organization, evidence/guidelines, clients’ preferences/values) when  implementing a practice change. | 1 2 3 4 5 6 7    Not competent Highly competent |
| 10. I participate in the process of stakeholder analyses (i.e., collecting and analyzing information on stakeholders’ importance and influence) when  implementing a practice change. | 1 2 3 4 5 6 7    Not competent Highly competent |

| 11. I participate in the development of an action plan to implement a practice change. | 1 2 3 4 5 6 7    Not competent Highly competent |
| --- | --- |
| 12. I participate in evaluating outcomes of evidence-informed decisions or practice changes. | 1 2 3 4 5 6 7    Not competent Highly competent |

Thank you for participating in our survey!

Please click **‘submit’** below to ensure your answers are submitted.

To support your ongoing development of competence in evidence-informed decision-making (EIDM), the [National Collaborating Centre for Methods and Tools](https://www.nccmt.ca/capacity-development/workshops-events) provides numerous learning resources and supports including: [online learning modules](https://www.nccmt.ca/learningcentre/EN/index.php), [webinars,](https://www.nccmt.ca/capacity-development/webinars) [assessment tools](https://www.nccmt.ca/capacity-development/skills-assessment-tool), [videos,](https://www.nccmt.ca/capacity-development/videos) [workshops and events.](https://www.nccmt.ca/capacity-development/workshops-events)
