# Supplementary material for: Validity, reliability, and acceptability of the Evidence-Informed Decision-Making (EIDM) competence measure
Source: PLoS One. 2022 Aug 5;17(8):e0272699. doi: 10.1371/journal.pone.0272699 (PMC9355195; doi:10.1371/journal.pone.0272699)
Supplement: S1 Table — (DOCX) [file pone.0272699.s002.docx]

**S1 Table. Low and high item-item correlations (40 items)**

| **Item** | **Item** | **Correlation** |
| --- | --- | --- |
| **Knowledge** | | |
| 1. Knowledge about ‘define’ step | 2. Knowledge about ‘search’ step | 0.845 |
| 3. Knowledge of evidence levels | 5. Knowledge of databases (pre-appraised evidence) | 0.812 |
| 3. Knowledge of evidence levels | 6. Knowledge about ‘appraise’ step | 0.815 |
| 6. Knowledge about ‘appraise’ step | 7. Knowledge of critical appraisal tools | 0.834 |
| 6. Knowledge about ‘appraise’ step | 9. Knowledge about ‘adapt’ step | 0.878 |
| 7. Knowledge of critical appraisal tools | 8. Knowledge about ‘synthesize’ step | 0.811 |
| 8. Knowledge about ‘synthesize’ step | 9. Knowledge about ‘adapt’ step | 0.936 |
| 8. Knowledge about ‘synthesize’ step | 10. Knowledge about ‘implement’ step | 0.829 |
| 8. Knowledge about ‘synthesize’ step | 11. Knowledge about ‘evaluate’ step | 0.810 |
| 9. Knowledge about ‘adapt’ step | 10. Knowledge about ‘implement’ step | 0.913 |
| 9. Knowledge about ‘adapt’ step | 11. Knowledge about ‘evaluate’ step | 0.810 |
| 10. Knowledge about ‘implement’ step | 11. Knowledge about ‘evaluate’ step | 0.884 |
| **Skills** | | |
| 1. Develop answerable question | 2. Develop search strategy | 0.906 |
| 1. Develop answerable question | 6. Assess applicability of evidence to local context | 0.826 |
| 2. Develop search strategy | 3. Use online databases (individual research studies) | 0.836 |
| 2. Develop search strategy | 4. Use online databases (synthesized research) | 0.862 |
| 2. Develop search strategy | 6. Assess applicability of evidence to local context | 0.839 |
| 2. Develop search strategy | 7. Conduct barrier/facilitator assessment | 0.824 |
| 3. Use online databases (individual research studies) | 4. Use online databases (synthesized research) | 0.888 |
| 4. Use online databases (synthesized research) | 6. Assess applicability of evidence to local context | 0.805 |
| 5. Use critical appraisal tools | 6. Assess applicability of evidence to local context | 0.812 |
| 6. Assess applicability of evidence to local context | 7. Conduct barrier/facilitator assessment | 0.909 |
| 6. Assess applicability of evidence to local context | 8. Conduct stakeholder analysis | 0.833 |
| 6. Assess applicability of evidence to local context | 9. Develop action plan | 0.853 |
| 7. Conduct barrier/facilitator assessment | 8. Conduct stakeholder analysis | 0.890 |
| 7. Conduct barrier/facilitator assessment | 9. Develop action plan | 0.900 |
| 8. Conduct stakeholder analysis | 9. Develop action plan | 0.923 |
| **Attitudes** | | |
| 1. Can implement EIDM in efficient way | A6 - believe use of guidelines improves practice/policy | 0.262 |
| 1. Can implement EIDM in efficient way | A7 - EIDM is difficult | 0.272 |
| 2. Can engage others in addressing barriers | 6. Believe use of guidelines improves practice/policy | 0.290 |
| 2. Can engage others in addressing barriers | 7. EIDM is difficult | 0.234 |
| 3. Believe evaluating outcomes important | 7. EIDM is difficult | 0.174 |
| 5. Believe critical appraisal important | 7. EIDM is difficult | 0.099 |
| 6. Believe use of guidelines improves practice/policy | 7. EIDM is difficult | 0.038 |
| 3. Believe evaluating outcomes important | 4. Believe EIDM improves services or programs | 0.824 |
| 4. Believe EIDM improves services or programs | 5. Believe critical appraisal important | 0.810 |
| 4. Believe EIDM improves services or programs | 6. Believe use of guidelines improves practice/policy | 0.867 |
| 5. Believe critical appraisal important | 6. Believe use of guidelines improves practice/policy | 0.807 |
| **Behaviours** | | |
| 5. Critical appraisal of individual studies | 6. Critical appraisal of synthesized evidence | 0.901 |
| 5. Critical appraisal of individual studies | 7. Synthesis and interpretation of evidence/form recommendations | 0.853 |
| 6. Critical appraisal of synthesized evidence | 7. Synthesis and interpretation of evidence/form recommendations | 0.886 |
| 7. Synthesis and interpretation of evidence/form recommendations | 8. Integrate evidence from expertise, preferences, context | 0.801 |
| 8. Integrate evidence from expertise, preferences, context | 9. Assessment of barriers/facilitators | 0.866 |
| 9. Assessment of barriers/facilitators | 10. Participate in stakeholder analyses | 0.820 |
| 9. Assessment of barriers/facilitators | 11. Action planning | 0.823 |
| 10. Participate in stakeholder analyses | 11. Action planning | 0.810 |
| 11. Action planning | 12. Evaluating outcomes | 0.857 |

**Note: ≤0.30 = low; ≥0.80 = high**
